# The diagnostic levels of evidence of instrumented devices for measuring viscoelastic joint properties and spasticity; a systematic review

**DOI:** 10.1186/s12984-022-00996-7

**Published:** 2022-02-11

**Authors:** Levinia Lara van der Velden, Maaike Anna Catharina de Koff, Gerard Maria Ribbers, Ruud Willem Selles

**Affiliations:** 1 Department of Rehabilitation Medicine, Erasmus MC University Medical Center Rotterdam, Doctor Molewaterplein 40, 3015 GD Rotterdam, The Netherlands; 2 Rijndam Rehabilitation, Rotterdam, The Netherlands; 3 Department of Plastic and Reconstructive Surgery, Erasmus MC University Medical Center Rotterdam, Rotterdam, The Netherlands

**Keywords:** Stroke, Cerebral palsy, Robotic, Spasticity, Viscoelastic joint properties, Clinical added value

## Abstract

**Background:**

Many diagnostic robotic devices have been developed to quantify viscoelastic properties and spasticity of patients with upper motor neuron lesions. However, in clinical practice, subjective and nonvalid clinical scales are still commonly used. To understand the limited use of diagnostic robotic devices assessing viscoelastic joint properties and spasticity in clinical practice, we evaluate the diagnostic level of evidence of studies on these devices.

**Method:**

A systematic literature review was performed using multiple databases. Two of the authors independently screened all articles. Studies investigating human subjects diagnosed with stroke or cerebral palsy, measured with a mechanical device to assess viscoelastic joint properties and/or spasticity of an extremity. All articles were assigned a diagnostic level of evidence, which was established with a classification strategy based on the number of participants and the design of the study, from a Level 0 (less than 10 subjects) to a Level IV, reporting the long-term clinical consequences in daily care.

**Results:**

Fifty-nine articles were included. Most studies measured the upper limb (64%) in stroke patients (81%). The highest level of evidence found was Level IIa (53%); these studies correlated the test values of the robotic device with a clinical test or within subgroups. Level 0 (30%) and Level I (17%; determining the range of values of the robotic test) were also common. None of the studies tested their device for diagnostic accuracy (Level III), clinical added value (Level IV).

**Conclusion:**

The diagnostic evidence needed for implementing robotic devices in clinical practice is lacking. Our findings indicate that more effort should be invested in studying diagnostic accuracy (Level III) or added value for clinical care (Level IV); only these studies can provide clinicians with evidence that robotic devices have added value above the currently-used clinical scales.

**Supplementary Information:**

The online version contains supplementary material available at 10.1186/s12984-022-00996-7.

## Introduction

In patients with upper motor neuron disease, non-neural (i.e. altered tissue viscoelastic joint properties) and neural (i.e. improper muscle activation, caused by e.g. spasticity) properties may contribute to joint stiffness [1]. These impairments are common as over eighty-five percent of all patients with upper motor neuron disease suffer from a paretic upper or lower extremity resulting in pain, difficulty moving, or poor hygiene [2, 3]. However, since impairments in non-neural and neural joint properties require different treatments, reliable, valid, and user-independent assessment is needed to support clinicians in selecting treatment plans.

Frequently used clinical tools to measure viscoelastic joint properties (e.g. Modified Tardieu Scale (MTS) and goniometer) and spasticity (e.g. Modified Ashworth Scale (MAS) and Modified Tardieu Scale (MTS)) of an extremity are subjected to the biases inherent to human perception. For measuring spasticity with a clinical measurement tool such as the MAS, studies show only fair to good intra-rater agreement scores [4, 5]. Fleuren et al. [6], due to its poor reliability and validity, stated that the MAS should not be used to evaluate spasticity anymore. Further, viscoelastic joint properties and spasticity cannot be differentiated with clinical tools [5, 7], which hampers individually tailored treatment plans.

Robotic measurement tools have the potential of more quantitative, objective, operator-independent assessment of viscoelastic joint properties and spasticity by imposing controlled positions or forces and measuring a person's individual responses with sensors. For example, during a velocity-controlled passive stretch, force sensors can measure the joint-muscle resistance during the whole movement trajectory [8–10]; changes in resistance that result from involuntary muscle activation can be a measure of spasticity [11].

Robotic measurement tools are not commonly used in clinical practice [12]. This may be due to multiple reasons, such as high purchase costs, relatively complex ease of use, and the relatively long measurement time. Another reason may be that no evidence is available of studies investigating the clinical added value of these robotic measurement tools.

Different ways of studying a diagnostic instrument's potential or diagnostic value are possible, depending on the kind of research question. To convert the diagnostic question into the appropriate research design, Sackett and Haynes [13] suggested four relevant questions that need to be answered in four different phases. Phase 1 Question: Do test results in affected patients differ from those in normal individuals? Phase 2 Question: Are patients with certain test results more likely to have the target disorder? Phase 3 Question: Do test results distinguish patients with and without the target disorder among those in whom it is clinically sensible to suspect the disorder? Phase 4 Question: Do patients undergoing the diagnostic test fare better than similar untested patients? The type of questions provide an increasing detailed insight into the diagnostic accuracy of the test, with the last type of question measuring the ultimate value of a diagnostic test; the health outcomes resulting from the interventions that the diagnostic test results precipitate.

To understand the limited use of diagnostic robotic devices assessing viscoelastic joint properties and spasticity in clinical practice, this systematic review was performed to evaluate the diagnostic level of evidence of studies investigating these devices in patients with stroke or cerebral palsy (CP).

## Methods

### Search strategy

This systematic review was conducted following the Preferred Reporting Items for Systematic Reviews and Meta-Analyses (PRISMA) guidelines [14].

We included mechanically driven robotic devices which use objective measurement outcomes (e.g., force, position, additional electromyography (EMG)) to estimate contributions of viscoelastic joint properties and/or spasticity of an extremity. Viscoelastic joint properties [15, 16] encompass a reduction in range of motion and changes in joint mechanical properties such as stiffness and damping and are assumed to be the result of muscle shortening (muscle contractures) or intrinsic changes in muscle tissue due to an increase in collagen fibers in the cellular joint matrix [1, 5, 17]. Spasticity is defined by Lance et al. (1980) [18] as "…a motor disorder, characterised by a velocity-dependent increase in tonic stretch reflexes (muscle tone) with exaggerated tendon jerks, resulting from hyper-excitability of the stretch reflex as one component of the upper motor neuron (UMN) syndrome" or by Sanger et al. (2003) [19] as "…hypertonia in which 1 or both of the following signs are present: 1) resistance to externally imposed movement increases with increasing speed of stretch and varies with the direction of joint movement, and/or 2) resistance to externally imposed movement rises rapidly above a threshold speed or joint angle". For this paper, we included studies that used either of these definitions.

We included English-language articles published between January 1946 and October 2020. The search strategy was developed in cooperation with a university librarian and comprised the following databases: Embase, Medline ALL, Web of Science Core Collection, Cochrane Central Register of Controlled Trials and Google Scholar. The following keywords were included: Stroke, Cerebral palsy, Diagnostic, Robotics, and Measurement.

In addition, we used CoCites to find additional articles [20]. In CoCites, we used the included paper, which had the highest number of citations (one paper for stroke patients and one for CP) from the above-mentioned search strategy as the primary papers (query articles). Then, CoCites finds articles based on their co-citation frequency to identify articles that address the same content as the query article. Next, CoCites organizes the identified articles based on the frequency of all citations that cite or are cited by the query articles.

Furthermore, a manual search was performed in Pubmed to retrieve missing papers because some papers used specific names for their measurement devices in their title/abstract that were not included in the results of the previously-mentioned search strategies. The complete search strategy and its outcomes can be found in Additional file 1.

### Selection criteria

To be included, a study needed to meet both of two inclusion criteria: (i) involving human subjects with stroke or cerebral palsy; (ii) quantifying viscoelastic joint properties and/or spasticity of human extremities with a robotic device. As mentioned before, any method of identifying viscoelastic joint properties or spasticity was accepted, as long as it was measured with a mechanically driven device and the quantitative data was used for analysis. Systematic reviews and meta-analyses were excluded. Also, conference papers, unpublished articles, and studies that only used EMG as an outcome measurement without using force or position parameters were excluded.

### Method of level classification

The model of Sackett and Haynes [13] describes four phases of diagnostic research with corresponding questions to help convert a clinical diagnostic question into the appropriate research design. In the current review, we adapted this model to identify five different research study designs (Levels 0-IV, see Table 1) corresponding to the phases identified by Sackett and Haynes.

**Table 1:** Levels of diagnostic evidence of studies using a diagnostic device to determine viscoelastic joint properties or spasticity with a robotic device

Level 0	Including less than ten participants
Level I	Reporting range of values of test results in patients and/or healthy controls
Level II	Correlating the test results with a reference test, or analyzing change over time, or comparing test results of patients and controls with descriptive statistics and significance testing
Level III	Reporting statistical values of diagnostic accuracy, such as sensitivity or specificity, either comparing confirmed patients with reference test and controls (e.g. under ideal conditions, according to Phase II of Sackett and Haynes) or distinguishing within a suspected patient population (e.g. more realistic clinical setting, according to Phase III of Sackett and Haynes
Level IV	Evaluating the clinical consequences of using the device by evaluating the clinical added value of the test results in patients who were measured with the device

Studies with less than ten participants are classified as Level 0, as these small sample sizes do not allow generalization. The cutoff point of ten participants was a choice of the authors. Studies that determine the range of values for viscoelastic joint properties or spasticity in patients or healthy controls are scored as Level I. Studies that compare patients and controls with descriptive statistics or with significance testing (e.g. t-test for group differences) are also scored as Level I. Studies that correlate the outcomes with a reference test (e.g., the MAS), compare outcomes between patients and controls, or analyze change over time (e.g., pre-intervention and post-intervention), are quantified as Level II. Level III studies determine the ability of the test to discriminate subjects with and without abnormal viscoelastic properties or spasticity, measured with a reference test, and report diagnostic accuracy with statistical values such as sensitivity or specificity. Note that the difference between Level II and III is that III analyses the ability to distinguish between subjects (either patients and controls or within a patient population), while II only compares the values of groups. Level IV studies evaluate the clinical consequences of using the diagnostic device (e.g. clinical added value) by evaluating the outcomes of the patients measured with the device and patients not measured with the device. This should establish whether using these tests in clinical care lead to better outcomes.

#### Data Extraction

All articles were reviewed for title/abstract, and the articles that met the inclusion criteria were also screened for full text. For data extraction and analysis purposes, we followed the Cochrane research methodology [21]. Two authors (MK, LV) with backgrounds in clinical rehabilitation medicine independently screened all articles for title/abstract and full-text using EndNote X9. The two reviewers discussed disagreements in scoring the included articles. When the reviewers still disagreed after a second evaluation, a third author (RS) decided. With the same screening process, the level of evidence (Level selection) was assigned to all included articles. From all included articles, we extracted the studied population, the joint measured by the robotic device, the clinical measurement used as a reference, and if a control group was present.

Since our goal was to describe the level of evidence of the available literature and not to describe the accuracy itself (e.g., the sensitivity and specificity), we did not apply a quality assessment of the included studies.

## Results

### Study selection

**Fig. 1:**
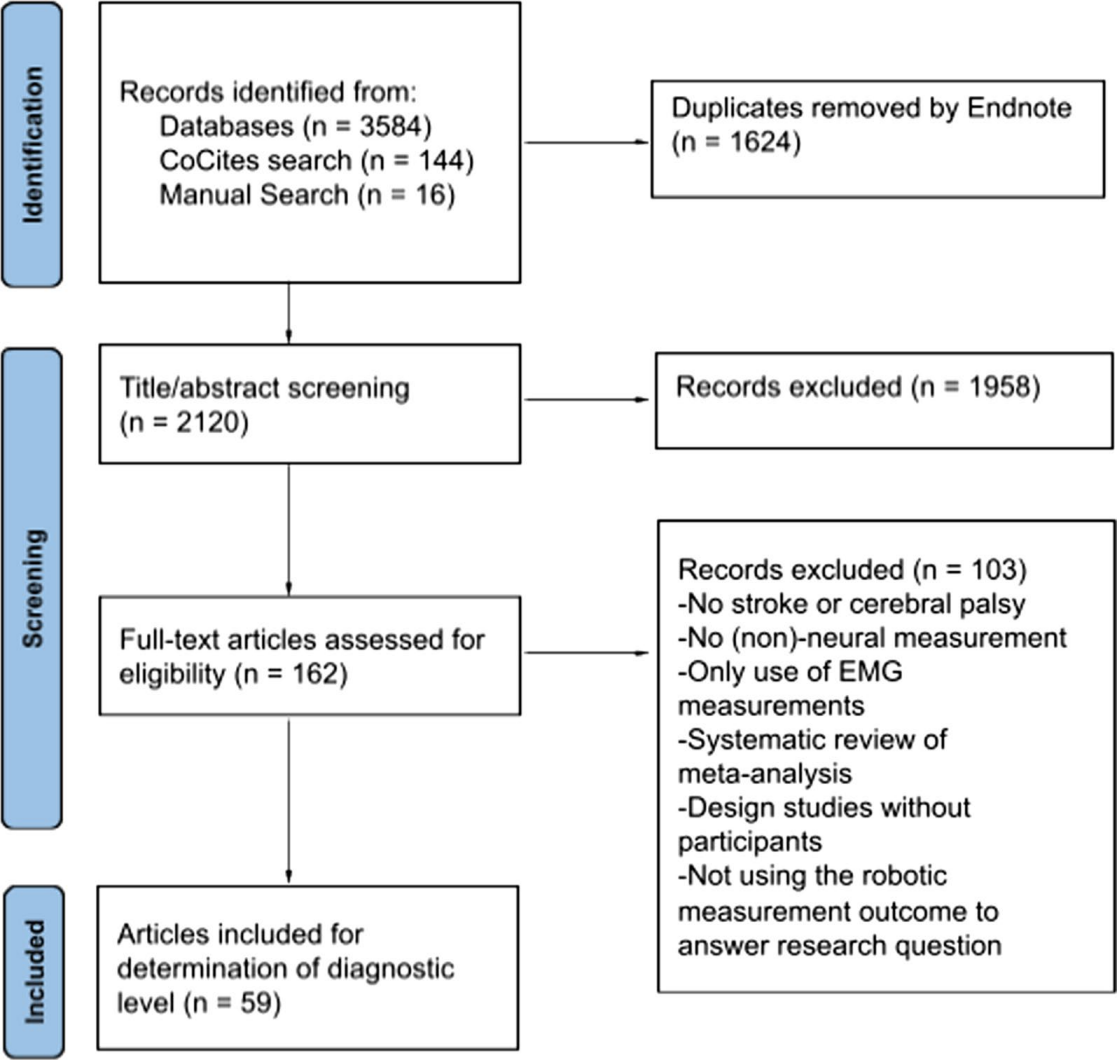
Flowchart article selection

The initial search strategy in databases yielded 3584 articles. With CoCites, an additional 144 articles were included, and the manual search added an additional 16 articles. After the entire selection process, a total of 59 articles (1.6%) were included in the systematic review (Fig. 1).

### Study characteristics

The characteristics of the included studies are shown in Additional file 2: Table S2, ordered by level of evidence. The total number of included patients was 1080; the mean sample size per study was 18 (± 18SD). In 42% of the studies, a control group was included. The total number of included controls was 504; the mean sample size per study was 9 (± 20SD). The majority of the studies measured adults with chronic stroke (68%); The other studies measured adults with subacute stroke (7%) and children with cerebral palsy (19%).

Most studies measured the upper extremities (64%); the ankle (30%), wrist (29%) and elbow (27%) were the most often measured joints. The most commonly-used reference test to quantify spasticity was the MAS (70%). For viscoelastic joint properties, all articles used the passive range of motion as a reference test. Many different robotic devices were used; the NeuroFlexor (15%) and the Wristalyzer (3%) were most commonly used. Both devices performed Level I and II.

The majority of the studies (51%) measured both the viscoelastic joint properties and spasticity. As outcomes, most studies quantified the torque–angle curve during movement (66%) and the passive range of motion (37%). A test–retest analysis to evaluate the reliability of the measurements was performed in 19% of the studies. It should, however, be noted that reliability may have been performed already in an earlier study, as was the case in some of the Neuroflexor studies (for example, see Andringa 2020 or Plantin 2019).

### Level characteristics

**Fig. 2:**
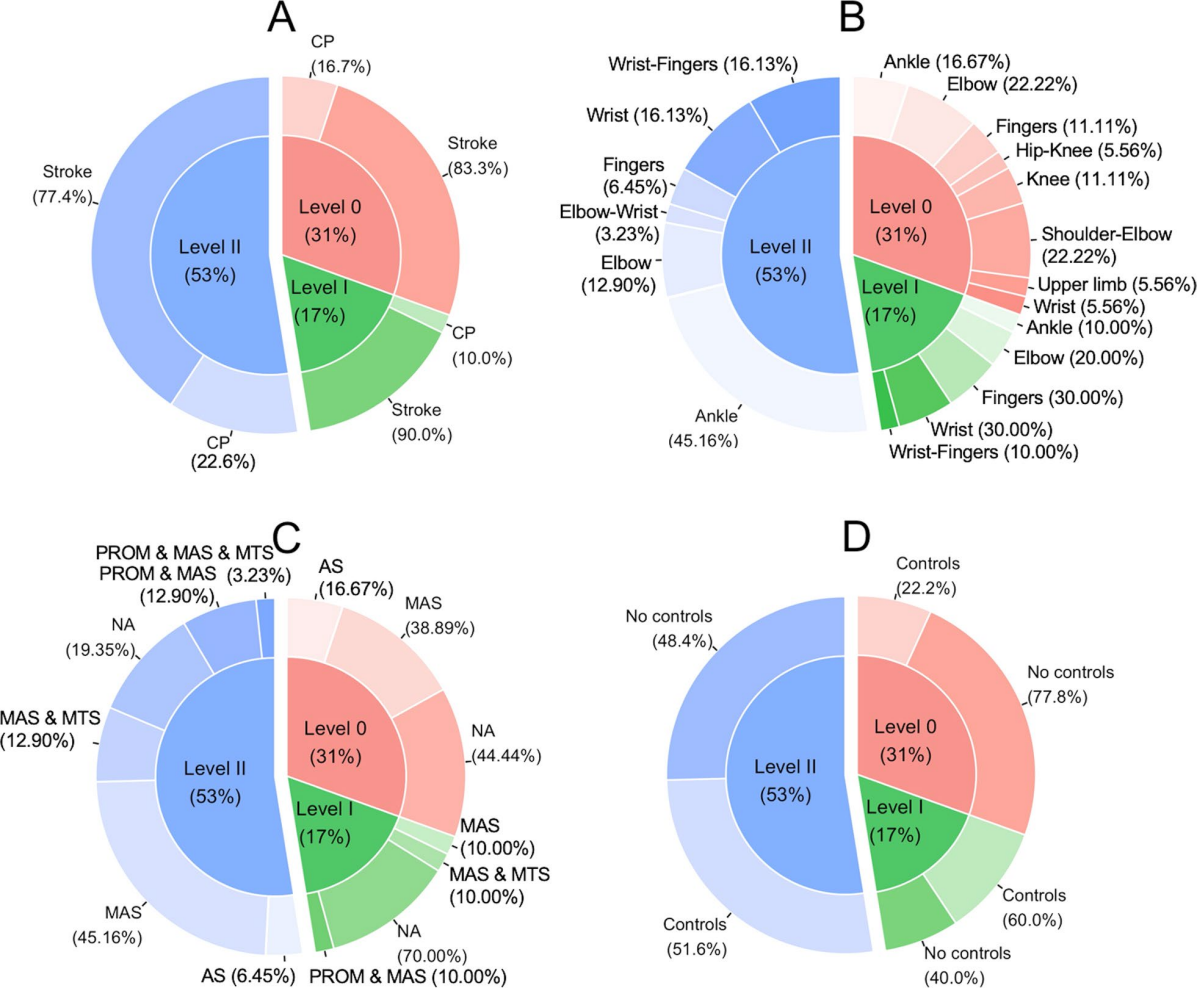
Distribution of the levels of evidence. The inner circles show the distribution in Levels. The donut around the pie in **A** illustrates the patient population that was measured, showing that most articles in all Levels measured adult stroke patients. The donut around the pie in **B** illustrates the measured joints, showing that most articles measured the shoulder in combination with the elbow in Level 0, the wrist or fingers in Level I and the ankle Level II. The donut around the pie in **C** illustrates the reference test, showing little variety in reference tests. The donut around the pie in **D** illustrates the number of articles that used control groups in certain levels. AS: Ashworth Scale, MAS: Modified Ashworth Scale, MTS: Modified Tardieu Scale, PROM: Passive Range of Motion, CP: Cerebral Palsy

Figure 2 shows the distribution of the different levels of evidence, together with the patient population (Fig. 2A), measured joints (Fig. 2B), reference tests (Fig. 2C) and control groups (Fig. 2D). The highest level was Level II (53% of the studies). Of the remaining studies, 30% were classified as Level 0, and 17% as Level I. None of the studies met the criteria for Level III or higher.

Studies classified as Level II had the largest sample size, with a mean of 25 (± 22SD) and measured most of the included children with cerebral palsy (23%). In studies classified as Level 0 and Level I an upper limb joint (67% respectively 90%) was most frequently measured, in comparison with studies with Level II in which the ankle (45%) was the most commonly measured joint. Control groups were mostly included in studies classified as Level I and Level II. In the studies classified as Level II, 81% used a reference test, compared to 30% in Level I and 56% in Level 0 studies.

## Discussion

This review shows that the diagnostic evidence needed for implementing robotic devices in clinical practice is lacking. We only found articles using research methods in diagnostic levels of evidence Level 0, I or II, for measuring viscoelastic joint properties and/or spasticity of patients with cerebral stroke, cerebral hemorrhage or cerebral palsy. None of the included studies tested their diagnostic robotic device for diagnostic accuracy (Level III) or added value (Level IV). To retrieve adequate evidence for the clinical implementation of diagnostic robotic devices, the higher levels of evidence need to be investigated.

One reason why the included studies were all classified as lower evidence levels could be that these are the first studies to perform in developing a new robotic device. Studies in Level 0, I or II designs have a role in initially showing the validity of the measurements. However, further studies are needed to determine the diagnostic accuracy of the device (Level III) or the added value (Level IV). Related to that, it was striking that the included studies mainly described newly developed robotic devices (81%) and only two devices (the NeuroFlexor [22] (Aggero MedTech AB, Älta, Sweden) and Wristalyzer [23]) were tested in multiple diagnostic levels. This may also reflect that many research groups in this field have a strong engineering background, focusing on developing hardware and software rather than on clinical testing. Translational research teams with both engineers and clinicians may be needed to overcome this problem.

A problem in clinical testing in this field is a lacking gold standard [4–7]. As stated in the introduction, clinical tools to measure viscoelastic joint properties and spasticity are subjected to the biases inherent to human perception. This is mainly a challenge for Level III study designs since these compare patients with and without the targeted disorder confirmed with a reference test. However, studying the impact of a diagnostic device (e.g. Level IV design) is possible without using a reference test, since it measures patient outcomes when using the device in clinical care by, for example, comparing patients measured with and without the device.

None of the included studies evaluated the impact of the diagnostic devices on clinical care (Level IV). An example of such a study, in this case comparing two different diagnostic workups for coronary artery disease, is the prospective diagnostic randomized clinical trial by M. Lubbers et al. [24] that compared the effectiveness and safety of a cardiac computed tomography (CT) algorithm with functional testing. An equivalent of such a study design for instrumented devices for measuring viscoelastic joint properties and spasticity could be a study where patients with a paretic limb with spasticity or abnormal viscoelastic properties seeking treatment are randomized to the diagnostic test or not. When the diagnostic test had added value then the test results could help the clinician to select the optimal treatment in individual patients, which should lead to better treatment outcomes than patients who were not tested.

While our aim was to describe the diagnostic level of evidence of the included studies, our review does provide an interesting overview of the characteristics of the robotic devices, joints most commonly studied and reference tests that were used. For example, we found that most studies measured a single joint; the majority of the articles measured the ankle or elbow. This might be explained by the fact that building a robotic device to measure one specific joint is less complex than a device to measure multiple joints [25]. Also, hinge joints such as the ankle or elbow joint are limited to two axes of movement and are less complex to measure than a multi-axial joint, such as the shoulder joint [26]. For clinical practice, this single-joint approach can be a problem since patients usually present with problems in different joints, which would currently require different robotic devices.

### Study limitations

A possible limitation of this study is that the level of evidence classification we used was not tested or validated. We based it on literature [13] and adjusted it to the type of studies performed, as described in the Methods. Also, we focused on studies that used kinetic measurements (e.g. force and angle positions), to specifically measure the concept of spasticity or viscoelastic joint properties, and not the functional measurements (e.g. Fugl-Meyer assessment, Action Research Arm Test or Box and Block test). A limitation is also that we aimed to classify the literature based on the level of evidence of the study design to identify potentially lacking types of studies; in this review, we did not set out to review the psychometric properties of the instruments (e.g. test–retest reliability, validity, responsiveness, sensitivity, specificity) nor to score the quality of the included studies answering the questions these studies aimed to answer.

## Conclusion/implications and recommendations for future research

This review shows a lack of diagnostic evidence to implement diagnostic robotic devices in clinical practice, since the highest level of evidence found was Level II. Therefore, this review emphasizes the importance of conducting further research with higher levels of diagnostic evidence to provide adequate evidence for reliable and valid implementation of the diagnostic robotic devices in clinical practice. To make a step towards implication in daily clinical care, existing devices with positive results in Level 0, I and II studies need to be extended in higher levels studies, which may require translational collaboration between clinicians, researchers, engineers and manufacturers to match the study methods with the diagnostic question optimally.

## Supplementary Information

**Additional file 1: Table S1.** Search string revision.

**Additional file 2: Table S2.** Study characteristics.

## Data Availability

The datasets used and/or analyzed during the current study are available from the corresponding author on reasonable request.

## Abbreviations

CP: Cerebral palsy
MAS: Modified Ashworth Scale
MTS: Modified Tardieu Scale
PRISMA: Preferred Reporting Items for Systematic Reviews and Meta-Analyses
EMG: Electromyography
UMN: Upper Motor Neuron
MK: Maaike de Koff
LV: Levinia van der Velden
RS: Ruud Selles
CT: Computed tomography
