# Supplementary material for: The diagnostic levels of evidence of instrumented devices for measuring viscoelastic joint properties and spasticity; a systematic review
Source: J Neuroeng Rehabil. 2022 Feb 11;19:16. doi: 10.1186/s12984-022-00996-7 (PMC8832664; doi:10.1186/s12984-022-00996-7)
Supplement: Supplementary file 1 — Additional file 1: Table S1. Search string revision. [file 12984_2022_996_MOESM1_ESM.pdf]

Additional file 1

(Update t.o.v. 20200116)

Table 1. Diagnostic robots cerebral palsy or stroke

Database searched	via	Years of coverage	References	After de-duplication
Embase	Embase.com	1971 - Present	1068	1027
Medline ALL	Ovid	1946 - Present	754	63
Web of Science Core Collection	Web of Knowledge	1975 - Present	1048	421
Cochrane Central Register of Controlled Trials	Wiley	1992 - Present	514	290
Other sources: Google Scholar			200	105
CoCites			93 (stroke) + 52(CP) = 145	143
Manual			16	16
**Total**			**3745**	**2120***

*Note: *Total of all references is not equal to the added number of each single database together in the file. This because it is an update of a previous search and an article can be removed/added from the used ranking or is removed/added from the database or the thesaurus terms of an article have been changed.*

***New references in EndNote file: 270 (t.o.v. 20200116)*

Embase.com

('cerebral palsy'/de OR 'cerebrovascular accident'/de OR 'lacunar stroke'/de OR 'stroke patient'/de OR (((cerebral*) NEAR/3 (pals*)) OR stroke* OR poststroke* OR cerebrovascular-accident* OR CVA):ab,ti,kw) AND ('robotics'/de OR 'machine'/de OR (robot* OR testing-machine* OR manipulator*):ab,ti,kw) AND ('diagnostic procedure'/de OR 'diagnosis'/de OR 'diagnostic test'/de OR 'measurement'/de OR 'biomechanics'/de OR 'prediction'/de OR 'neurologic disease assessment'/de OR 'disease assessment'/de OR 'neurologic examination'/de OR (diagnos* OR biomechanic* OR neuromechanic* OR robot*-based-parameter* OR (robot* NEAR/2 measurement*) OR predict* OR assessment* OR quantificat*):ab,ti,kw) NOT ((animal/exp OR animal*:de OR nonhuman/de) NOT ('human'/exp)) NOT ([Conference Abstract]/lim AND [1800-2018]/py)

Medline

(Cerebral Palsy/ OR Stroke/ OR Stroke, Lacunar/ OR (((cerebral*) ADJ3 (pals*)) OR stroke* OR poststroke* OR cerebrovascular-accident* OR CVA).ab,ti,kf.) AND (Robotics/ OR (robot* OR testing-machine* OR manipulator*).ab,ti,kf.) AND ("Diagnostic Techniques and Procedures"/ OR Diagnosis/ OR Diagnostic Techniques, Neurological/ OR Neurologic Examination/ OR (diagnos* OR biomechanic* OR neuromechanic* OR robot*-based-parameter* OR (robot* ADJ2 measurement*) OR predict* OR assessment* OR quantificat*).ab,ti,kf.) NOT (exp animals/ NOT humans/) NOT (news OR congres* OR abstract* OR book* OR chapter* OR dissertation abstract*).pt.

Web of Science

TS=(((((cerebral*) NEAR/2 (pals*)) OR stroke* OR poststroke* OR cerebrovascular-accident* OR CVA)) AND ((robot* OR testing-machine* OR manipulator*)) AND ((diagnos* OR biomechanic* OR neuromechanic* OR robot*-based-parameter* OR (robot* NEAR/2 measurement*) OR predict* OR assessment* OR quantificat*))) AND DT=(Article OR Review)

Cochrane CENTRAL

((((cerebral*) NEAR/3 (pals*)) OR stroke* OR poststroke* OR cerebrovascular NEXT accident* OR CVA):ab,ti,kw) AND ((robot* OR testing NEXT machine* OR manipulator*):ab,ti,kw) AND ((diagnos* OR biomechanic* OR neuromechanic* OR (robot NEXT based NEXT parameter*) OR (robot* NEAR/2 measurement*) OR predict* OR assessment* OR quantificat*):ab,ti,kw)

Google Scholar *Top 200 relevant references*

“cerebral palsy|palsies”|stroke|“cerebrovascular accident”|CVA robot|robots|robotic diagnosis|diagnoses|biomechanics|neuromechanics|“robot|robots|robotic measurement|prediction|assessment|quantification”

CoCites

Two most cited articles (highest number of citations) from the databases above were used; one for CP and one for Stroke. These articles were used as the query articles as input for the search system of CoCites: https://www.cocites.com/
