# Supplementary material for: The diagnostic levels of evidence of instrumented devices for measuring viscoelastic joint properties and spasticity; a systematic review
Source: J Neuroeng Rehabil. 2022 Feb 11;19:16. doi: 10.1186/s12984-022-00996-7 (PMC8832664; doi:10.1186/s12984-022-00996-7)
Supplement: Supplementary file 2 — Additional file 2: Table S2. Study characteristics. [file 12984_2022_996_MOESM2_ESM.pdf]

Additional file 2

Table 2. Study characteristics

Author (year)	Level	Diagnosis	Phase stroke	Patients/ controls (n)	Age group	Reference test	Measured joints	Device name	Measurement	Measurement outcome	Test retest
Piovesan et al. (2011)	0	Stroke	Chronic	9/0	Adult	AS	Shoulder + elbow	Braccio di Ferro	Spasticity	Resistance torque	No
Stienen et al. (2011)	0	Stroke	NA	1/0	NA	NA	Elbow	ACT-4D HapticMaster	Spasticity	Torque + EMG	No
Piovesan et al. (2011)	0	Stroke	Chronic	9/0	Adult	AS	Shoulder + elbow	Braccio di Ferro	Spasticity + Viscoelastic joint properties	Torque Angle Reaction	No
Zadravec et al. (2014)	0	Stroke	Chronic	1/0	Adult	MAS	Elbow	Haptic Master robot system	Spasticity	Torque Angle Reaction	No
Godfrey et al. (2010)	0	Stroke	Chronic	4/0	Adult	AS	Fingers	HEXORR	Viscoelastic joint properties	Torque Angle Reaction + PROM	No
Ren et al. (2013)	0	Stroke	Chronic	3/2	Adult	NA	Shoulder + elbow + wrist	IntelliArm	Viscoelastic joint properties	PROM	No
Schmartz et al. (2011)	0	CP	NA	9/0	Child	NA	Hip + knee	L-STIFF	Spasticity + Viscoelastic joint properties	Torque Angle Reaction	Yes
Palazzolo et al. (2007	0	Stroke	NA	1/0	Elderly	MAS	Shoulder + elbow	MIT-MANUS	Spasticity	Impedance	No
Posteraro et al. (2018)	0	Stroke	Chronic	5/0	Adult	MAS	Elbow	NEUROExos Elbow Module system	Spasticity	Torque Angle Reaction	No

Author (year)	Level	Diagnosis	Phase stroke	Patients/ controls (n)	Age group	Reference test	Measured joints	Device name	Measurement	Measurement outcome	Test retest
Zhoa et al. (2011)	0	CP	NA	7/0	Child	NA	Ankle	No Name	Viscoelastic joint properties	Torque Angle Reaction	No
Zhang et al. (2002)	0	Stroke	Chronic	4/5	Adult	NA	Ankle	No Name	Spasticity + Viscoelastic joint properties	Torque Angle Reaction + PROM + EMG	No
Lerner et al. (2019)	0	CP	NA	6/0	Child	MAS	Knee	No Name	Spasticity	Stretch-reflex velocity gain	No
Park et al. (2008)	0	Stroke	Chronic	4/0	Adult	NA	Elbow	No Name	Spasticity + Viscoelastic joint properties	Torque Angle Reaction	No
Kung et al. (2016)	0	Stroke	Chronic	9/0	Adult	MAS	Shoulder + elbow	No Name	Viscoelastic joint properties	Force Angle Reaction + Passive ROM	No
Zhou et al. (2016)	0	Stroke	Chronic	7/7	Adult	NA	Ankle	PKU-RARS	Viscoelastic joint properties	Torque Angle Reaction + PROM	No
Ranzani et al. (2019)	0	Stroke	Chronic	5/5	Adult	MAS	Fingers	ReHapticKnob	Viscoelastic joint properties	Torque Angle Reaction	No
Kim et al. (2013)	0	Stroke	Chronic	1/0	Adult	NA	Knee	Walkbot system	Viscoelastic joint properties	Torque Angle Reaction	No
Baniasad et al. (2011)	0	Stroke	Chronic	1/0	Adult	MAS	Wrist	Wrist-RoboHa b	Spasticity	Torque Angle Reaction	No
McPherson et al. (2019)	I	Stroke	Chronic	13/0	Adult	MAS	Elbow	Biodex Rehabilitation testing system	Spasticity + Viscoelastic joint properties	Torque Angle Reaction	No

Author (year)	Level	Diagnosis	Phase stroke	Patients/ controls (n)	Age group	Reference test	Measured joints	Device name	Measurement	Measurement outcome	Test retest
Xu et al. (2020)	I	CP	NA	17/17	Child	NA	Ankle	IntelliStretch	Spasticity + Viscoelastic joint properties	Torque Angle Reaction + EMG	No
Wang et al. (2017)	I	Stroke	Chronic	17/17	Adult	NA	Wrist	Neuroflexor	Spasticity + Viscoelastic joint properties	Resistance torque	No
Gaverth et al. (2013)	I	Stroke	Chronic	34/10	Adult	PROM + MAS	Wrist + fingers	Neuroflexor	Spasticity + Viscoelastic joint properties	Resistance torque	Yes
Kamper et al. (2000)	I	Stroke	Chronic	13/2	Adult	NA	Fingers	No Name	Spasticity + Viscoelastic joint properties	Torque Angle Reaction + PROM + EMG	No
Kamper et al. (2003)	I	Stroke	Chronic	12/1	Adult	NA	Fingers	No Name	Spasticity	Torque Angle Reaction; + EMG	No
Kamper et al. (2006)	I	Stroke	Chronic	30/0	Adult	NA	Fingers	No Name	Spasticity + Viscoelastic joint properties	Torque Angle Reaction + EMG	No
Sin et al. (2019)	I	Stroke	Chronic	17/0	Adult	MAS + MTS	Elbow	No Name	Spasticity	Torque Angle Reaction + EMG	Yes
de Gooijer-Van de Groep et al. (2018)	I	Stroke	Subacute	36/0	Adult	NA	Wrist	Wristalyzer	Spasticity + Viscoelastic joint properties	Torque Angle Reaction	No
Van der Krogt et al. (2015)	I	Stroke	Chronic	32/14	Adult	NA	Wrist	Wristalyzer	Spasticity + Viscoelastic joint properties	Torque Angle Reaction + ROM + reflex loop time	Yes

Germanotta et al. (2020)	II	Stroke	Subacute	120/0	Adult	MAS	Fingers	Amadeo	Spasticity	Torque Angle Reaction + PROM	Yes
Roy et al. (2011)	II	Stroke	Chronic	10/20	Adult	MAS + ROM	Ankle	Anklebot	Viscoelastic joint properties	Torque Angle Reaction	No
Starsky et al. (2005)	II	Stroke	Chronic	16/0	Adult	MAS	Elbow	Biodex Multi-Joint System 3B	Spasticity + Viscoelastic joint properties	Torque Angle Relation	Yes

Author (year)	Level	Diagnosis	Phase stroke	Patients/ controls (n)	Age group	Reference test	Measured joints	Device name	Measurement	Measurement outcome	Test retest
Centen et al. (2017)	II	Stroke	Chronic	46/96	Adult	MAS + MTS	Elbow	KINARM robotic exoskeleton	Spasticity + Viscoelastic joint properties	Torque Angle Reaction + PROM + release after catch	No
Leng et al. (2019)	II	Stroke	Subacute	15/0	Adult	NA	Wrist	Neuroflexor	Spasticity + Viscoelastic joint properties	Resistance torque	No
Wang et al. (2018)	II	Stroke	Chronic	21/0	Adult	NA	Wrist	Neuroflexor	Spasticity + Viscoelastic joint properties	Resistance torque	No
Gaverth et al. (2014)	II	Stroke	Chronic	22/0	Adult	PROM + MAS	Wrist + fingers	Neuroflexor	Spasticity + Viscoelastic joint properties	Resistance torque	No
Wu et al. (2010)	II	CP	NA	12/0	Child	MAS	Ankle	No Name	Viscoelastic joint properties	Torque Angle Reaction + PROM	No
Chen et al. (2014)	II	CP	NA	12/0	Child	MAS + MTS	Ankle	No Name	Viscoelastic joint properties	PROM	No
Willerslev-Olsen et al. (2013)	II	CP	NA	35/28	Child	MAS + MTS	Ankle	No Name	Spasticity + Viscoelastic joint properties	Torque response to stretch + EMG	No
Gao et al. (2011)	II	Stroke	Chronic	10/10	Adult	NA	Ankle	No Name	Viscoelastic joint properties	Torque Angle Reaction	No
Waldman et al. (2013)	II	Stroke	Chronic	12/12	Adult	MAS	Ankle	No Name	Viscoelastic joint properties	PROM	No
Chen et al. (2016)	II	CP	NA	41/0	Child	MAS	Ankle	No Name	Viscoelastic joint properties	PROM	No

Author (year)	Level	Diagnosis	Phase stroke	Patients/ controls (n)	Age group	Reference test	Measured joints	Device name	Measurement	Measurement outcome	Test retest
Chung et al. (2004)	II	Stroke	Chronic	24/32	Adult	MAS	Ankle	No Name	Viscoelastic joint properties	Torque Angle Reaction + PROM	No
Selles et al (2005)	II	Stroke	Chronic	10/0	Adult	NA	Ankle	No Name	Spasticity + Viscoelastic joint properties	Torque Angle Reaction + PROM	No
Lorentzen et al. (2010)	II	Stroke	Chronic	10/31	Adult	AS	Ankle	No Name	Spasticity + Viscoelastic joint properties	Torque Angle Reaction + ROM + EMG	No
de Vlugt et al. (2010)	II	Stroke	Chronic	19/7	Adult	AS	Ankle	No Name	Spasticity + Viscoelastic joint properties	Torque Angle Reaction + ROM	Yes
Nam et al. (2017)	II	Stroke	Chronic	20/1	Adult	MAS	Elbow + wrist	No Name	Spasticity	Torque Angle Reaction	Yes
Fischer et al. (2007)	II	Stroke	Chronic	15/0	Adult	NA	Fingers	No Name	Spasticity + Viscoelastic joint properties	Torque Angle Reaction + PROM	No
Chen et al. (2014)	II	CP	NA	23/0	Child	MAS	Ankle	No Name	Spasticity + Viscoelastic joint properties	PROM	No
Xiadoyan et al. (2017)	II	Stroke	Chronic	14/0	Adult	MAS	Wrist	No Name	Spasticity + Viscoelastic joint properties	Torque Angle Reaction + PROM	No
Wu et al. (2011)	II	CP	NA	12/0	Child	MAS + MTS	Ankle	No Name	Viscoelastic joint properties	Torque Angle Reaction	No
Wu et al. (2010)	II	CP	NA	12/0	Child	MAS	Ankle	No Name	Viscoelastic joint properties	Torque Angle Reaction + PROM	No

Lum et al. (1999)	II	Stroke	Chronic	13/6	Adult	NA	Elbow	PUMA 260 robot	Spasticity	Torque Angle Reaction	Yes
Dehem et al. (2017)	II	Stroke	Chronic	12/0	Adult	MAS	Elbow	REAplan robot	Spasticity	Torque Angle Reaction	No
De Gooijer-Van de Groep et al. (2016)	II	Stroke	Chronic	30/14	Adult	MAS	Wrist	Wristalyzer	Spasticity + Viscoelastic joint properties	Torque Angle Reaction + ROM	No
Andringa, A. et al (2020)	II	Stroke	Acute	17/17	Adult	MAS	Wrist + fingers	Neuroflexor	Spasticity + Viscoelastic joint properties	Resistance torque	Yes
Lindberg, P.G. (2011)	II	Stroke	Chronic	31/13	Adult	MAS	Wrist	No Name	Spasticity + Viscoelastic joint properties	Force Angle Reaction + EMG	No
Pennati, G.V. et al (2016)	II	Stroke	Acute	39/107	Adult	MAS + PROM	Wrist + fingers	Neuroflexor	Spasticity + Viscoelastic joint properties	Resistance torque	Yes
Andringa, A. et al (2019)	II	Stroke	Chronic	45/30	Adult	MAS + PROM + MTS	Wrist + fingers	Neuroflexor	Spasticity + Viscoelastic joint properties	Resistance torque	Yes
Plantin, J. et al (2019)	II	Stroke	Subacute	55/0	Adult	PROM + MAS	Wrist + fingers	Neuroflexor	Spasticity + Viscoelastic joint properties	Resistance torque	Yes

Abbreviations: CP: cerebral palsy; NA: not available; AS: Ashworth Scale; MAS: Modified Ashworth Scale; MTS: Modified Tardieu Scale; (P)ROM: (Passive) Range Of Motion; EMG: Electromyography. Note: Phase stroke: acute 0–7 days; subacute 7 days–3 months; chronic >6 months Age group: child <12 years; young adult 12–18 years; adult 18–65 years; elderly >65 years.
